# Erythrocyte-derived extracellular vesicles transcytose across the blood-brain barrier to induce Parkinson’s disease-like neurodegeneration

**DOI:** 10.1186/s12987-025-00646-9

**Published:** 2025-04-14

**Authors:** Hélèna L. Denis, Aurélie de Rus Jacquet, Melanie Alpaugh, Michel Panisset, Roger A Barker, Éric Boilard, Francesca Cicchetti

**Affiliations:** 1 Centre de recherche du CHU de Québec, Axe Neurosciences, T2-07 2705, Boulevard Laurier, Québec, QC, G1V 4G2 QC Canada; 2 Département de Psychiatrie & Neurosciences, Université Laval, Québec, QC Canada; 3 Department of Molecular and Cellular Biology, University of Guelph, Guelph, Canada; 4 Centre Hospitalier de l’Université de Montréal and Centre de recherche du Centre Hospitalier de l’Université de Montréal, Département de neuroscience, Université de Montréal, Montréal, QC Canada; 5 Department of Clinical Neurosciences, John van Geest Centre for Brain Repair, University of Cambridge, Cambridge, UK; 6 Département de microbiologie et immunologie, Université Laval, Québec, QC Canada

**Keywords:** Parkinson’s disease, Erythrocytes, Extracellular vesicles, Blood brain barrier, 3D model

## Abstract

Parkinson’s disease (PD) is a neurodegenerative illness characterized by motor and non-motor features. Hallmarks of the disease include an extensive loss of dopaminergic neurons in the *substantia nigra pars compacta*, evidence of neuroinflammation, and the accumulation of misfolded proteins leading to the formation of Lewy bodies. While PD etiology is complex and identifying a single disease trigger has been a challenge, accumulating evidence indicates that non-neuronal and peripheral factors may likely contribute to disease onset and progression. The brain is shielded from peripheral factors by the blood-brain barrier (BBB), which tightly controls the entry of systemic molecules and cells from the blood to the brain. The BBB integrates molecular signals originating from the luminal (blood) and abluminal (brain) sides of the endothelial wall, regulating these exchanges. Of particular interest are erythrocytes, which are not only the most abundant cell type in the blood, but they also secrete extracellular vesicles (EVs) that display disease-specific signatures over the course of PD. Erythrocyte-derived EVs (EEVs) could provide a route by which pathological molecular signals travel from the periphery to the central nervous system. The primary objective of this study was to evaluate, in a human-based platform, mechanisms of EEV transport from the blood to the brain under physiological conditions. The secondary objective was to determine the ability of EEVs, generated by erythrocytes of healthy donors or patients, to induce PD-like features. We leveraged two in vitro models of the BBB, the transwell chambers and a microfluidic BBB chip generated using human induced pluripotent stem cells. Our findings suggest that EEVs transcytose from the vascular to the brain compartment of the human BBB model via a caveolin-dependant mechanism. Furthermore, EEVs derived from individuals with PD altered BBB integrity compared to healthy EEV controls, and clinical severity aggravated the loss of barrier integrity and increased EEV extravasation into the brain compartment. PD-derived EEVs reduced ZO-1 and Claudin 5 tight junction levels in BMEC-like cells and induced the selective atrophy of dopaminergic neurons. In contrast, non-dopaminergic neurons were not affected by treatment with PD EEVs. In summary, our data suggest that EEV interactions at the human BBB can be studied using a highly translational human-based brain chip model, and EEV toxicity at the neurovascular unit is exacerbated by disease severity.

**Supplementary Information:**

The online version contains supplementary material available at 10.1186/s12987-025-00646-9.

## Highlights

Erythrocytes secrete extracellular vesicles that can transcytose into the brain via a caveolin-dependant mechanism.

A microfluidic brain chip can be used to evaluate mechanisms of transcytosis across the blood-brain barrier.

The clinical severity of Parkinson’s disease affects how erythrocyte-derived extracellular vesicles interact with cerebral endothelial cells.

Erythrocyte-derived extracellular vesicles generated from donors with Parkinson’s disease alter the blood-brain barrier and induce atrophy of dopaminergic neurons.

**Supplementary Information:**

The online version contains supplementary material available at 10.1186/s12987-025-00646-9.

## Introduction

Parkinson’s disease (PD) is a neurodegenerative disorder of complex etiology, owing to the contribution of both genetic and environmental factors as potential disease triggers. Neuropathology in the central nervous system (CNS) is, in large part, characterized by an extensive loss of dopaminergic neurons of the *substantia nigra pars compacta*, along with dopaminergic denervation of the striatum leading to significant movement impairments in affected individuals. Other cardinal features include neuroinflammation and aggregation of the α-synuclein (α-Syn) protein into inclusions known as Lewy bodies [1]. Additionally, emerging evidence suggests reduced blood-brain barrier (BBB) integrity in patients, which is associated with dysfunction of efflux pumps (e.g. p-glycoprotein) and an abnormal parenchymal accumulation of serum proteins [2–6]. Morphological and structural changes have also been reported in post-mortem PD tissue, including lower vascular density in the *substantia nigra* [3]. The molecular mechanisms implicated in BBB weakening remain to be fully elucidated, but reduced Zonula Occludens-1 (ZO-1) and occludin tight junction levels in postmortem cerebellum tissue of individuals with PD has been documented [7]. The investigation of PD-related preclinical in vivo models suggests reduced coverage of brain capillaries by astrocytic end-feet in the cortex and striatum, and diminished occludin protein levels in the striatum [2]. Furthermore, brain chips produced using human cells suggested that inflammatory PD astrocytes could compromise the BBB [3] and, in another study, α-Syn preformed fibrils incubated in the brain compartment of a microfluidic model increased paracellular permeability across the endothelium [8]. However, the brain vasculature is a structure consisting of a luminal (blood) and an abluminal (parenchyma) side, and each of these components has the potential to independently affect BBB function. Therefore, it can be envisioned that alterations of blood-brain communication via a dysfunctional BBB could be further aggravated (or induced) by disease-related changes to plasma composition. To support this concept, studies have shown PD-specific modifications to plasma profiles [9–13] and, while the main disease hallmarks involve pathological alterations within the CNS, there is a strong rationale to investigate how the periphery plays a critical role in PD onset and progression [14, 15].

The entry of circulating factors into the brain is tightly regulated by endothelial tight junctions and transporters, which selectively enable or prevent protein transcytosis from the blood circulation to the parenchyma. Circulating proteins are found as soluble factors, bound to carriers or enclosed within extracellular vesicles (EVs), which are membrane-bound entities produced by most cell types and secreted into the extracellular environment as a means of autocrine or paracrine communication. EVs are of particular interest as they are known to package signaling molecules including lipids, genetic material, and proteins, and can induce a biological response in healthy [16] or PD-afflicted [17] recipient cells. EVs secreted by erythrocytes (EEVs) are of specific relevance, as these blood elements have been suggested to contain a proteomic signature that can differentiate healthy controls from people with PD [18], and they may be able to propagate disease-related signals [19]. Indeed, it has been demonstrated that EEVs derived from healthy subjects can cross the BBB in rodent models, and EEVs derived from PD donors can reach the parenchyma where they induce microglial reactivity [20]. In essence, EEVs are complex biological vectors that carry signals, may serve as biomarkers, and mediate cell-to-cell communication across the organism. It is therefore essential to understand the mechanisms by which EEVs reach the healthy human brain, and evaluate whether EEV transport through the vasculature in disease conditions could affect BBB integrity and contribute to the loss of BBB function reported in patients with PD [4–6].

We leveraged a humanized microfluidic model of the BBB to recapitulate blood-brain interactions in vitro, and we recently demonstrated the relevance of this approach to model the PD BBB [3]. This platform relies on the differentiation of induced pluripotent stem cells (iPSCs) into cell types of the neurovascular unit and introduces fluid sheer stress to promote the self-organization of iPSC-derived endothelial cells into a three-dimensional vessel. Using both cell culture monolayers and the brain chip model, we provide evidence that brain-microvascular endothelial (BMEC)-like cells internalize EEVs via caveolin-mediated endocytosis, and EEVs derived from healthy donors transcytose across the bioengineered human neurovasculature. Furthermore, PD EEVs incubated in the 3D vasculature reduced BBB integrity, and EEVs isolated from patients of advanced clinical stages generated higher toxicity. In addition, PD EEVs induced dopaminergic neuronal atrophy, further confirming their relevance to PD pathology.

## Materials and methods

### Ethics statement and participant recruitment

Institutional review boards approved this study (CHU de Québec, #A13-2-1096; CHUM, #14.228; Cambridge Central Regional Ethics Committee, REC #03/303 & #08/H0306/26; and Cambridge University Hospitals Foundation Trust Research and Development department, R&D #A085170 & #A091246) in accordance with the Declaration of Helsinki, and written informed consent was obtained from all participants. Donors were asked to fill out a questionnaire related to health issues and medication, and a full blood count was performed on the day of blood sampling. The collection of blood samples was conducted by the same team of investigators, following identical procedures in both the UK and Canada. The UK Parkinson’s Disease Society Brain Bank criteria were used to diagnose PD patients, which gives a diagnostic accuracy of 98.6% when performed by movement disorder specialists [21, 22]. At the time of blood collection, patients underwent additional clinical evaluation using the Unified Parkinson Disease Rating Scale (UPDRS), which enabled classification into mild (*n* = 6, UPDRS = 29±4, 3 males/3 females, mean age = 59±4) and severe PD (*n* = 6, UPDRS = 80±10, 3 males/3 females, mean age = 68±5). The donors were selected to represent an equal number of male and female participants, and we focused our experiments on mild and severe PD cases to compare early and late disease stages.

**Fig. 1:**
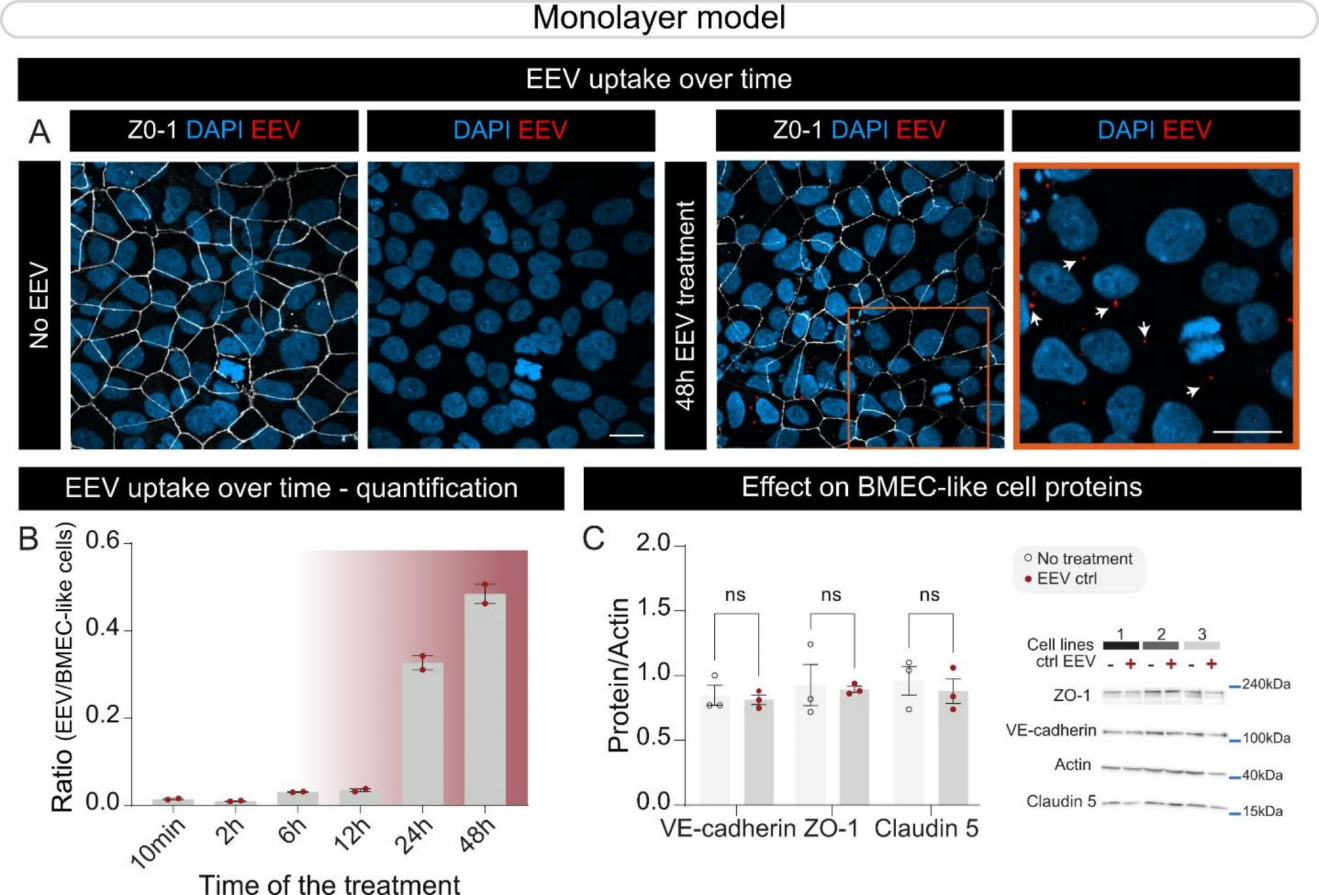
Human BMEC-like cells internalize EEVs. (**A-B**) Representative confocal images of control BMEC-like cells stained for ZO-1 (white), DAPI nuclear stain (**A**), and time-course quantification of EEV internalization (CellMask Deep red, white arrows) (**A-B**) from 10 min to 48 h incubation times. Scale bars: 20 μm. (**C**) Western blot-based quantification of tight junction protein levels for VE-cadherin, ZO-1, and Claudin 5 in BMEC-like cell monolayers after a 48 h incubation with EEVs. Protein levels are normalized to actin loading control. *Statistical analysis*: Error bars represent mean + SEM. Statistical analysis was performed using two-way ANOVA followed by Bonferroni’s multiple comparisons test. In (**B**), two different iPSC lines (line IDs 38554 and 41658) and in (**C**), three different iPSC lines (line IDs 38554, 41658, and 36091) were treated with EEVs produced from erythrocytes of two different healthy donors (age = 30 years old). *Abbreviations*: Ctrl, control; DAPI, 4′,6-diamidino-2-phenylindole; EEV, extracellular vesicles derived from erythrocytes; h, hours; min, minutes; ns, not significant; ZO-1, Zonula occludens-1

**Fig. 2:**
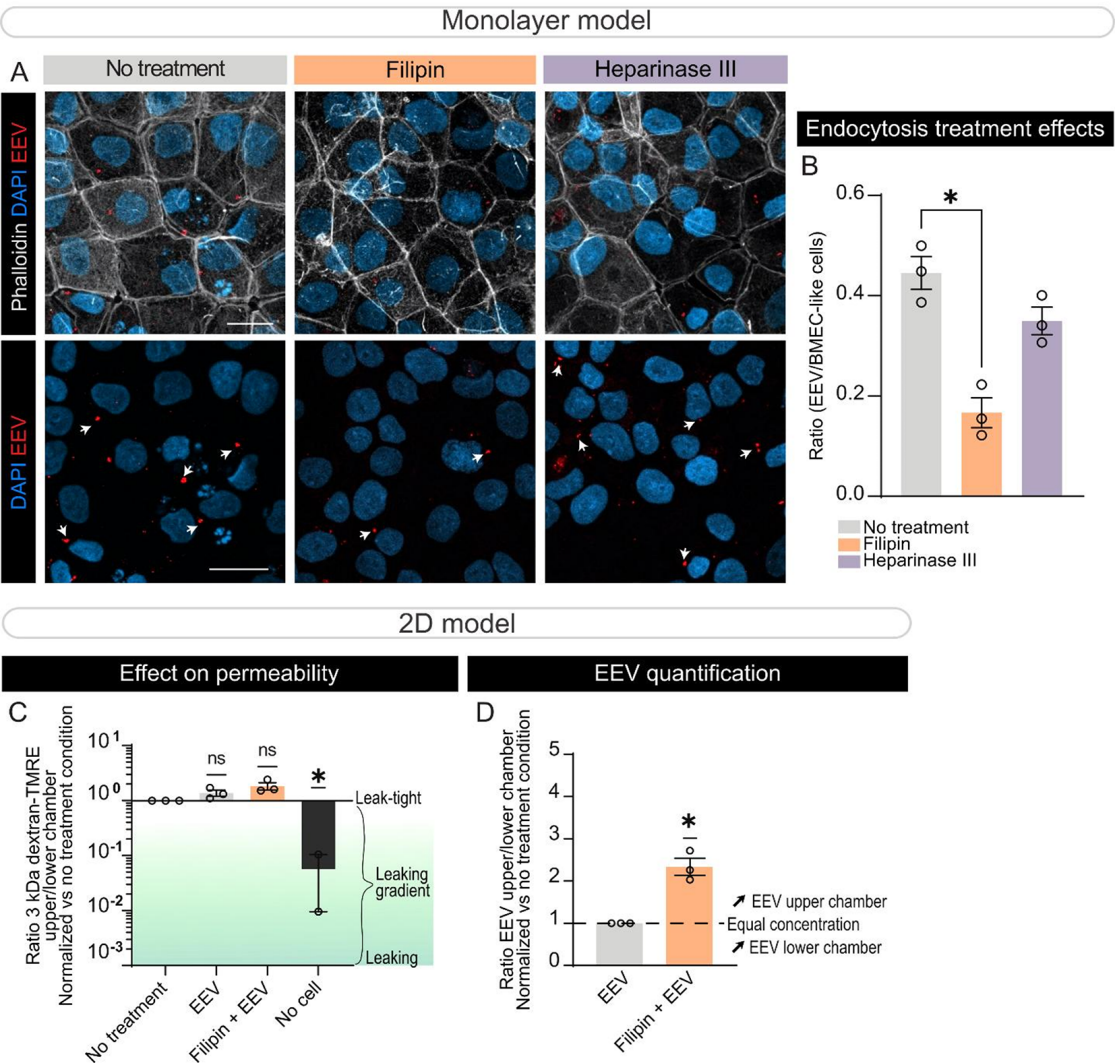
Caveolin-mediated endocytosis is implicated in EEV uptake by BMEC-like cells. (**A-B**) Representative confocal images of control BMEC-like cells stained for phalloidin (white) and DAPI nuclear stain, showing internalized EEVs (CellMask orange, white arrows) (**A**), and corresponding quantification of the number of internalized EEVs per BMEC-like cell (**B**) when the cultures were exposed to EEVs in the presence or absence of 5 µM Filipin or 6 mUI/mL heparinase III. Scale bars: 20 μm. (**C-D**) Nanoparticle FACS-based quantification of EEV transcytosis (**C**) and measure of monolayer integrity (**D**) in the transwell model when the BMEC-like cell growth medium located in the top chamber is supplemented with control EEVs, EEVs + 5 µM Filipin or vehicle control, absence of EEVs, or absence of BMEC-like cells (a positive control for 3 kDa dextran-TMRE penetration into the bottom compartment). *Statistical analysis*: Error bars represent mean + SEM. Statistical analysis was performed using one-way ANOVA followed by Tukey’s multiple comparison test (*p-value < 0.05; **p-value < 0.01) (**B**), or Wilcoxon signed rank test with theoretical median (untreated) set at 1 (*p-value < 0.05) (**C-D**). In this figure, a biological replicate consists of BMEC-like cells treated with EEVs that were produced from two different healthy donors (age = 30 years old) and data is averaged to generate a single value. Data points show three biological replicates collected using three different iPSC lines (line IDs 38554, 41658 and IM2-GC). *Abbreviations*: EEV, extracellular vesicles derived from erythrocytes

**Fig. 3:**
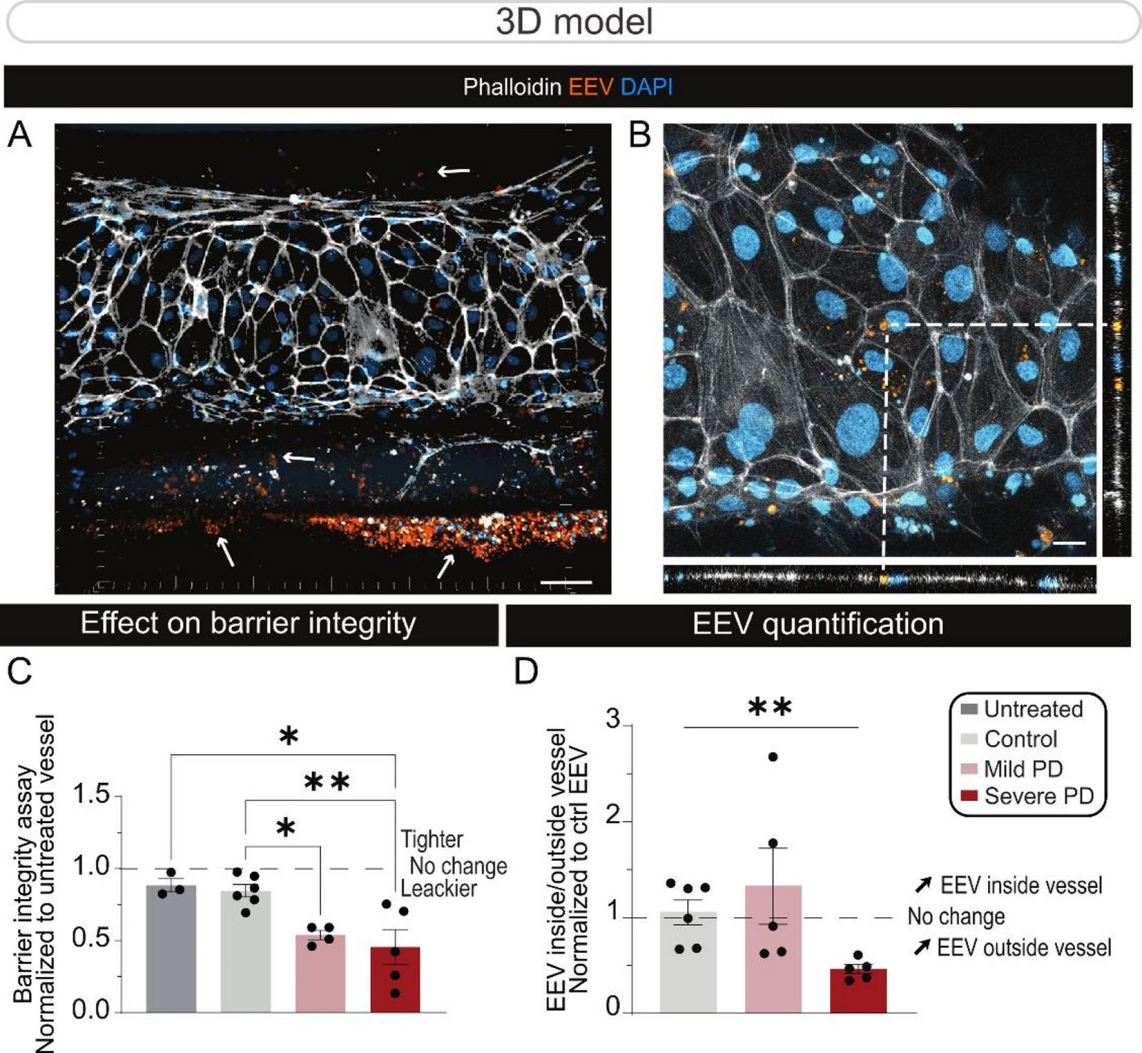
PD-EEVs cross the human BBB chip. (**A-B**) Confocal images of the endothelial vessel in the BBB chip showing BMEC-like cells (phalloidin, white) and the presence of EEVs (CellMask orange) in the abluminal side of the vessel (white arrows) (**A**) and within the endothelial cells (**B**) 48 h post-treatment. Scale bars: 50 μm (**A**), 20 μm (B). (**C**) Quantification of 3 kDa dextran-TMRE apparent permeability (P_aap_) values in BBB chips treated with vehicle control (i.e. untreated), control EEVs, mild or PD EEVs. (**D**) Nanoparticle FACS-based quantification of EEVs in conditioned media collected from the vascular or brain compartments. EEVs quantified in the brain compartment have transcytosed from within the luminal side of the endothelial vessel. In (**C**) and (**D**), each point represents a control vessel (line ID #38554) untreated (*n* = 3) or treated with EEVs from control subjects (*n* = 6 different donors, mean age = 68±5), or patients with mild (*n* = 4–5 different donors, UPDRS = 29±4, mean age = 59±6) or severe (*n* = 5 different donors, UPDRS = 80±10, mean age = 67±7) clinical manifestations according to the UDPRS scale. *Statistical analysis*: Error bars represent mean + SEM. Statistical analysis was performed using Wilcoxon signed rank test with theoretical median (**C** – untreated; **D** – Ctrl EEV) set at 1 (*p-value < 0.05; **p-value < 0.01). *Abbreviations*: Ctrl, control; DAPI, 4′,6-diamidino-2-phenylindole; EEV, extracellular vesicles derived from erythrocytes; PD, Parkinson’s disease

**Fig. 4:**
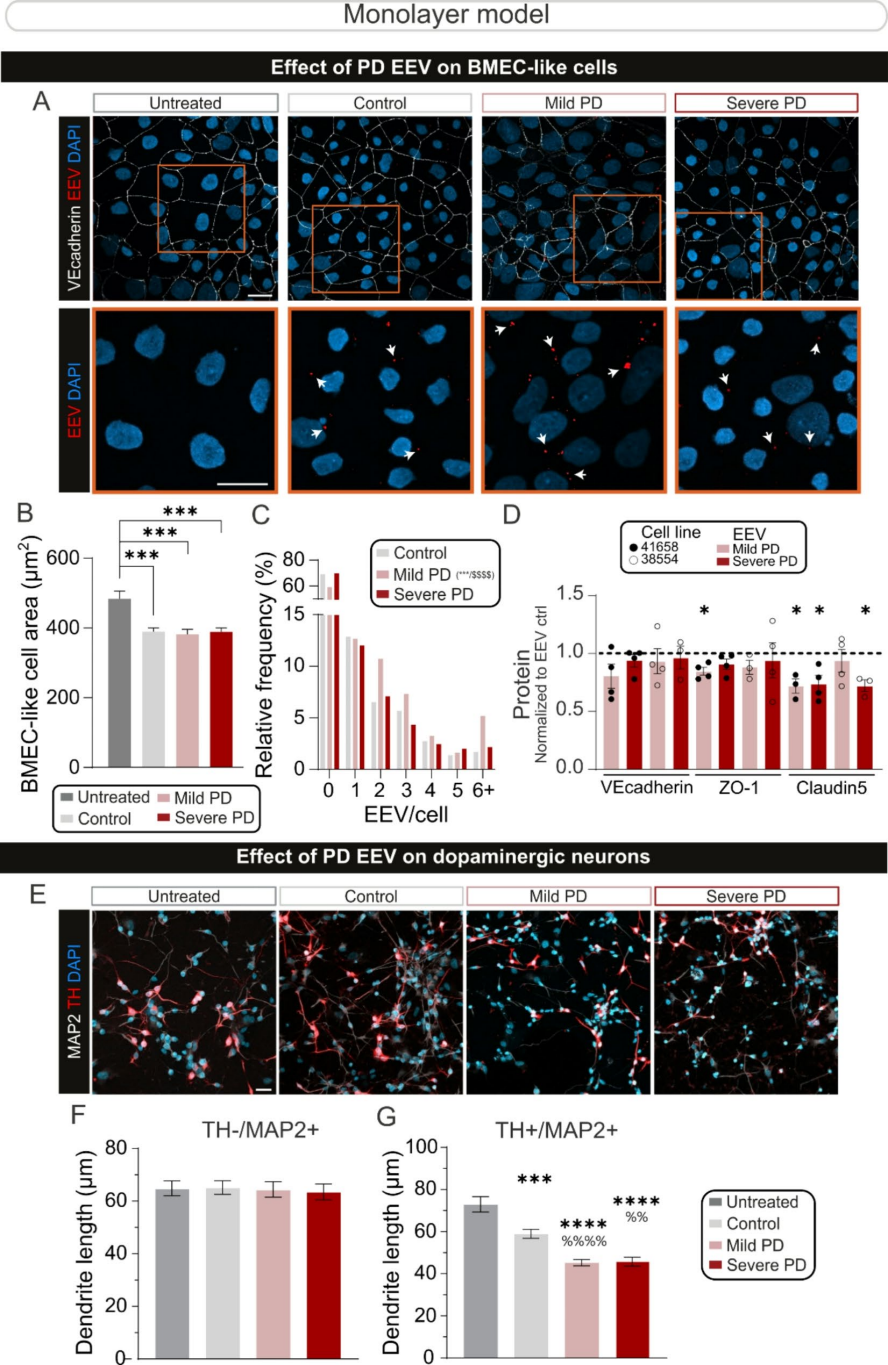
PD-EEVs reduce tight junction levels in BMEC-like cells and induce dopaminergic neuron atrophy. (**A**) Representative confocal images of control BMEC-like cells immunostained for VE-cadherin (white), internalized EEVs are shown in red (red, white arrows) and DAPI nuclear stain is shown in blue. Scale bar: 20 μm. (**B-C**) Quantification of BMEC-like cell area (**B**) and frequency distribution (**C**) in untreated conditions or after 48 h of treatment with EEV derived from control, mild PD or severe PD. (**D**) Western blot-based quantification of tight junction protein levels for VE-cadherin, ZO-1, and Claudin 5 in BMEC-like cell monolayers after a 48-h incubation with EEVs. Protein levels are normalized to BMEC-like cell treated with EEV control. (**E**) Representative confocal images of iPSC-derived neurons labeled for the pan-neuronal marker MAP2 (white) and a marker specific for dopaminergic neurons (TH, red). Images were taken after a 72-h treatment with vehicle control (i.e. untreated), age-/sex-matched control EEVs, mild or severe PD EEVs. Scale bar: 25 μm. (**F-G**) Quantification of MAP2^+^/TH^−^ (**F**) and TH^+^/MAP2^+^ (**G**) neurite lengths when iPSC-derived neurons are incubated in the absence or presence of EEVs derived from PD donors diagnosed with mild or severe PD, or age-/sex-matched controls, as shown in (**E**). *Demographics*: control subjects (*n* = 2–4, mean age = 70±4), patients with mild (*n* = 2–4, UPDRS = 26±3, mean age = 60±3) or severe (*n* = 4, UPDRS = 90±8, mean age = 68±5) PD. *Statistical analysis*: Error bars represent mean + SEM. Statistical analysis was performed using One-way ANOVA followed by Tukey’s multiple comparisons test: Comparison vs. untreated ***p-value < 0.001, ****p-value < 0.0001; Comparison vs. control EEV ^%%^p-value < 0.01, ^%%%%^p-value < 0.001 (**B**,** F-G**); Kuskal-Wallis test followed by Dunn’s multiple comparisons test: Comparison vs. untreated ***p-value < 0.001, ****p-value < 0.0001; Comparison vs. PD severe EEV p-value < 0.0001 (**C**). Statistical analysis was performed using Wilcoxon signed rank test with theoretical median (**D** – Ctrl EEV) set at 1 (*p-value < 0.05). (**B-C**) Corresponds to the BMEC-like area (**B**) or EEV/BMEC-like cell (**C**) of two independent replicates where two different iPSC donors (line IDs 38554 and 41658) and EEVs from two different individuals per group were used (i.e. at least 300 cells/experimental group); (**D**) Corresponds to two different iPSC donors (line IDs 38554 and 41658) and EEVs sampled from three to four different individuals per group were used. (**F-G**) Corresponds to the combined neurite length values of two independent replicates where two different iPSC donors (line IDs 38554 and IM2-GC) and EEVs from two different donors per group were used (i.e. at least 150 neurites/experimental group). *Abbreviations*: DAPI, 4′,6-diamidino-2-phenylindole; EEV, extracellular vesicles derived from erythrocytes; MAP2, microtubule-associated protein 2; ZO-1, Zonula occludens-1

Experiments shown in Figs. 1, 2 and S1 were performed using blood samples collected from two different healthy female controls (age 25 and 39 years old) at CHU de Québec, with no clinical reports of co-morbidities or medication intake. Experiments shown in Figs. 3 and 4 were performed using blood samples collected from 12 PD [Cambridge UK, *n* = 6 males and 6 females, mean age = 63±5] and 6 age- and sex-matched healthy controls [CHUM, *n* = 3 males and 3 females, mean age = 69±5].

### Production, purification and labelling of EEVs

Blood samples from control and PD donors were assigned a sample ID and all laboratory analyses were blinded to participant status. Production and purification of EEVs was performed as previously reported [18]. Briefly, blood was collected in heparin tubes and centrifuged for 10 min (min) at 282 x g at room temperature (RT). The cell pellet was then washed in phosphate buffered saline (PBS) supplemented with 2% fetal bovine serum (FBS), followed by a 0.9% sodium chloride solution, and centrifuged for 10 min at 750 x g. To avoid leukocyte and/or platelet contamination, the buffy coat and upper fraction of erythrocytes were removed. To cryopreserve erythrocytes, two volumes of glycerolyte 57 solution (57% glycerol, 142 mM sodium lactate, 1 mM KCl, 25 mM sodium phosphate pH 6.8) were added to the pellet and stored at -80 °C.

For the in vitro generation of EEVs, cryopreserved red blood cell samples were thawed and cultured as described previously [18] and, to visualize EEVs, erythrocytes were labelled using CellMask Deep Red plasma membrane stain (ThermoFisher, #C10046) according to the manufacturer’s protocol (Figures S1A, B). Briefly, erythrocytes were stained using a 0.5X staining solution for a total of 10 min at 37 °C, followed by two washes in 150mM NaCl to remove excess membrane stain. The erythrocyte pellet was then activated with 3 volumes of calcium ionophore solution (150 mM NaCl; 10 mM Tris-HCl; 3 mM CaCl_2_; 10 µM calcium ionophore A23187 (SigmaAldrich, # C7522)) for 1,5 h (h) at 37 °C. Activation was then stopped by adding 5 mM EDTA, and erythrocytes were pelleted at 2,500 x g for 20 min. The supernatant containing EEVs was then transferred into an Amicon 100 kDa Ultra-4 column (Millipore, #Z740198-96EA) and washed twice using filtered PBS 1X at 3,000 x g for 15 min. Approximately 25 µL of concentrated and purified EEVs were obtained and quantified using flow cytometry, as described previously [18]. The EEV sample was stored at 4 °C and used for experiments within one week. BMEC-like cell monolayers and BBB chip vessels were treated with a concentration of 2 × 10^6^ EEVs/mL, which corresponds to the mean EEV concentration observed in the blood of human donors [18]. Neuronal cultures were treated with an EEV concentration corresponding to the mean EEV concentration measured in the brain compartment of the BBB chips after a 48-h perfusion of the vessel (control condition: 1.10^6^ EEVs/mL).

### Nanoparticle fluorescence-activated cell sorting (FACS) quantification

EEV concentrations were quantified using a FACS Canto II Special Order Research Product equipped with a forward scatter (FSC) coupled to a photomultiplier tube (FSC-PMT) and a small particle detector. FACS performance tracking was carried out daily using the BD cytometer setup and tracking beads (BD Biosciences, San Jose, CA, USA). Settings for EEV detection were determined using a threshold of 200 for side scatter (SSC), as described previously [18, 23]. Fluorescently labeled erythrocytes or EEVs (using Cell-Mask Deep Red – see previous section) were counted using the filter for allophycocyanin (APC) (Figure S1). For all experiments, PBS was filtered on 0.22 μm pore size membranes (Millipore, #SLGVVR255F) to remove potential particulate contaminants that may confound data acquisition. EEV signal acquisition was performed at low speed at an approximate injection rate of 10 µl/min. To validate the specific detection of vesicular EEV particles, samples were incubated for 30 min with 0.1% Triton X-100, and analyzed using the nanoparticle FACS. Under these conditions, EEV membranes are ruptured while protein aggregates are left intact [24], and loss of EEV signal indicates the specific detection of vesicular structures (Figure S1C-E).

### Cell culture

#### iPSCs

Four independent patient-derived iPSC lines were used in this study. Two control iPSC lines were obtained via the NINDS repository (ND 41658, male; ND 38554, female), one control iPSC line was kindly provided by Dr. Randall Moon (female; line ID 36091) [17], and a fourth control iPSC line was generously provided by Prof. Dr. Thomas Gasser (Universitätsklinikum Tübingen) and Prof. Dr. Hans R. Schöler (Max-Planck Institute) (female, line ID IM2-GC) [25]. iPSCs were maintained in mTesR Plus medium (StemCell Technologies, # 100–0276), passaged as small aggregates using ReLeSR (StemCell Technologies, # 100–0483) and plated onto Geltrex-coated culture dishes (Thermo Fisher Scientific, #A14133).

#### Cell culture and differentiation of BMEC-like cells, dopaminergic neurons, and astrocytes

*BMEC-like cells.* IPSCs (line IDs 41658, 38554, IM2-GC, 36091) were used to prepare human BMEC-like cells. They were passaged and maintained in TeSR™ E8 medium (StemCell Technologies, #05990), and differentiated into BMEC-like cells before each experiment, as previously described [3]. Prior to differentiation, iPSCs were dissociated into single cells using Accutase (Millipore Sigma, #A6964) and plated at a density of 12,500 cells/cm^2^ on Geltrex coated plates in E8 medium supplemented with ROCK inhibitor (Y-27632, StemCell Technologies, #72302) (Day -1). After 24 h, the cells were cultured in TeSR™ E6 medium (StemCell Technologies, #05946) with daily media changes for 4 days (Days 0–3). On Days 4 and 5, media was substituted to endothelial cell (EC) medium (human endothelial serum-free medium, Thermo Fisher Scientific, #11111044; 1% platelet-poor human plasma (Sigma, #P2918); 20 ng/mL FGFb (StemCell Technologies, #78003); 10 µM retinoic acid, Sigma, #R2625)). On Day 6, the cells were harvested in Accutase and plated on Geltrex-coated dishes or in BBB microfluidic chips for experiments in EC + medium (i.e. EC medium supplemented with 20 µM RO-20-1724, 400 µM dibutyryl cAMP and 10 µM retinoic acid). For experiments related to Fig. 2, BMEC-like cells were treated with 5 µM filipine (Sigma, #F4767) or 6 mUI/mL of Heparinase III (Sigma, #H8891) for 30 min at 37ºC prior to EEV exposure.

*Dopaminergic neurons and astrocytes.* The differentiation of iPSCs into dopaminergic neurons was performed as reported previously [17, 26]. Briefly, iPSCs (line IDs IM2-GC and 38554) were first neuralized by dual SMAD inhibition, and the resulting midbrain-patterned neural progenitor cells (NPCs) were differentiated into dopaminergic neurons by incubating NPCs in neuronal differentiation medium for 7 to 10 days. Neuron differentiation medium consisted of neurobasal medium (Thermo Fisher, #21103-049) supplemented with 2% (v/v) B27 supplement (minus vitamin A; Thermo Fisher, #12587010), 100 U/ml penicillin-streptomycin (Thermo Fisher, #15140122), 1% (v/v) GlutaMAX (Thermo Fisher, #35050061), 0.2 mM ascorbic acid (Sigma, #A92902), 0.5 mM dibutyryl cAMP (Millipore Sigma, #28745), 10 µM DAPT (Tocris Bioscience, #2634), 20 ng/ml recombinant human BDNF (PeproTech, #450-02), 20 ng/ml recombinant human GDNF (PeproTech, #450 − 10), and 1 ng/ml recombinant human TGF-β3 (R&D Systems, #8420-B3-025). This protocol generates ~ 70% tyrosine hydroxylase (TH)-expressing cells that are considered dopaminergic neurons. The remaining ~ 30% that express pan-neuronal markers, and do not express TH, are not considered dopaminergic neurons [17, 26].

To generate astrocytes, the midbrain-patterned NPCs were differentiated as per previous publication [3]. Briefly, iPSCs (line ID 38554) were first neuralized by dual SMAD inhibition to generate midbrain-patterned NPCs, which were subsequently differentiated into astrocytes by culturing and subpassaging cells with astrocyte medium (ScienCell Research Laboratories, #1801) for a total of four weeks. Then, the astrocytes were used in cell-based assays or cryopreserved for long-term storage. Protocol validation was published previously and indicated expression of astrocyte markers CD44 and vimentin, and 100% of the iPSC-derived astrocytes expressed GFAP [17, 27].

#### 2D transwell BBB model

BMEC-like cells were differentiated from 3 control iPSC cell lines (36091, 38554, 41658), as described above. The cells were harvested in Accutase and plated on Geltrex-coated 12 mm transwell inserts (VWR, #29442-080) in EC + media supplemented with 10 µM ROCK inhibitor. The next day, BMEC-like cells were treated for 48 h with EEVs derived from healthy controls in the presence or absence of 5 µM of Filipine (Sigma, #F4767) or 6 mUI/mL of Heparinase III (Sigma, #H8891). Integrity of the BMEC-like cell layer was assessed before and after EEV treatment by replacing the growth medium with EC + supplemented with 500 µg/mL 3 kDa dextran-TMRE (Invitrogen, #D3308) for 12 h. Media was subsequently collected from the top and bottom chambers, and fluorescence intensity was measured using a microplate reader (BioTek Synergy). The EEV-containing media from the top and bottom chambers was additionally analyzed using nanoparticle FACS to quantify EEV concentration.

#### 3D microfluidic chip BBB model

OrganoPlate (Mimetas, Gaithersburg, MD) were used to prepare 3D models of the BBB on individual tissue culture chips as previously performed [3]. Each chip consists of a top lane used to grow the endothelial-like vessel, a middle lane filled with an ECM gel and a bottom lane plated with human primary pericytes and iPSC-derived astrocytes. Primary human brain vascular pericytes (ScienCell Research Laboratories, #1200) were cultured in complete pericyte medium (ScienCell Research Laboratories, #1201), as recommended by the manufacturer [3]. IPSC-derived astrocytes were differentiated and cultured as detailed in the *Cell Culture* section above.

The day prior to cell plating, the middle perfusion lane of the Organoplate 3-lane 40 was loaded with collagen I (7.5 mg/mL, Corning, #354236) and the vascular perfusion lane was coated with a combination of collagen IV (330 µg/mL, Sigma-Aldrich, #C6745) and fibronectin (50 µg/mL, Sigma-Aldrich, #F0895). On the day of plating, cells were harvested and plated into each microfluidic lane (*vascular channel*: 140,000 BMEC-like cells; *brain channel*: coculture of 15,000 astrocytes and 15,000 pericytes) [3]. The formation of endothelial-like vessels in the BBB chips was carried out for 6 days, followed by the intraluminal perfusion of EEVs in EC + medium. After 2 days of EEV treatment, conditioned media from each microfluidic lane was collected to conduct FACS-based quantification of EEV transcytosis from the vascular to the brain compartment. In addition, a barrier integrity assay was performed before and at the end of the treatment period [3]. Briefly, EC media was supplemented with 500 µg/mL 3 kDa dextran-TMRE (Invitrogen, #D3308). Fluorescence intensity representing dye migration from the vascular to the brain compartment was quantified using ImageJ (version 1.53 T) and apparent permeability (P_app_) values were calculated [3].

### Immunofluorescence

*Monolayers.* Cells grown on Geltrex-coated glass coverslips were washed once with DPBS and fixed in 4% paraformaldehyde (Fisher Scientific, #50-276-33) for 10 min at RT, washed twice with DPBS, and then blocked/permeabilized in blocking buffer (0.3% TX-100, 1% BSA, 10% FBS in DPBS) for 1 h at RT. Cells were then incubated overnight at 4 °C in BSA buffer (1% BSA in PBS) using Phalloidin-Atto 488 (Sigma, #49409) or incubated with a primary antibody according to the cell type of interest. BMEC-like cells were incubated with anti-VE-cadherin antibody (goat, 1:500, R&D Systems, #AF938) or anti-ZO-1 (rabbit, 1:500, Invitrogen, #617300), and neurons were incubated with anti-MAP2 (rabbit, 1:1000, Proteintech, #174-90-1-AP) and anti-TH (mouse, 1:500, Sigma Aldrich, #MAB318) antibodies. The following day, the cells were washed twice with DPBS and incubated in Alexa-conjugated secondary antibodies of the appropriate species (donkey anti-goat Alexa Fluor 488, #A11055; donkey anti-rabbit Alexa Fluor 555, #A31572; donkey anti-mouse Alexa Fluor 488, #A21202; Thermo Fisher Scientific) diluted 1:500 in BSA buffer for 1 h at RT. The cells were then washed three times with DPBS prior to a 7-min incubation with a DAPI nuclear stain (1:5,000, Molecular Probes, #D3571). Lastly, the cells were washed twice with DPBS and mounted onto glass slides using Fluoromount (Sigma Aldrich, #F4680).

*BBB microfluidic plate.* Cultures in the microfluidic chips were washed once with DPBS and fixed in 4% paraformaldehyde (Fisher Scientific, #50-276-33) for 20 min at RT. The chips were then washed once with DPBS, blocked/permeabilized in saponin buffer (0.1% saponin, 1% BSA, 10% FBS in DPBS) for 1 h at RT, and incubated overnight at 4 °C using Phalloidin-Atto 488 (Sigma, #49409). The next day, nuclei were stained using DAPI (1:5,000 dilution, Molecular Probes, #D3571) for 15 min at RT. Cells were washed three times with DPBS and stored at 4 °C.

### Protein quantification by Western blot

BMEC-like cells grown as monolayers were lysed using 1X RIPA buffer (Cell Signaling Technologies, #9806S) supplemented with 1X Halt protease and phosphatase inhibitor cocktail (Thermo FisherScientific, #78440). Protein concentration was quantified using a BCA assay (Thermo Fisher Scientific, #23250) and samples were mixed with sample buffer (Laemli 1X, Biorad, #161–0747) before heating at 90 °C for 5 min. A total of 20 µg of protein was migrated on a 3–8% tris-acetate gel for one hour and 45 min at 100 V, and then transferred onto a 0.45 μm polyvinylidene difluoride membrane (GE Healthcare Life Science: 10600023) overnight at 20 V followed by a boost to 100 V for 20 min. All membranes were washed in PBS-Tween 0.1% and blocked by preincubation in a solution of 5% skimmed milk, 0.5% bovine serum albumin (BioShop Canada: ALB001) for one hour. Membranes were subsequently incubated overnight at 4 °C with primary antibodies in blocking solution: anti-GAPDH (anti-mouse, 1:5,000, Abm, #G041), anti-actin (mouse, 1:5,000 dilution, Abm, #G043), anti-Claudin5 (rabbit, 1:500 dilution, Millipore, #ABT45), anti-VE-cadherin (rabbit, 1:1,000 dilution, Abcam, #ab33168), anti-ZO-1 (rabbit, 1:500 dilution, Thermofisher, #61-7300). The next day, membranes were washed with PBS-Tween 0.1% and incubated with the corresponding secondary antibody conjugated with horseradish peroxidase goat anti-mouse (1:25,000 dilution, Jackson ImmunoResearch, #115-035-166) for one hour at RT. Membranes were visualized using myECL imager (Thermo Fisher Scientific) after a 2-min incubation in chemiluminescence reagent (Immobilon Luminata Forte, EMD, Millipore, #WBLUF0500). Semi-quantitative analyses of the western blots were performed using MYImageAnalysis software in which membranes were analyzed and band intensities were expressed as a ratio of protein of interest against Actin or GAPDH (loading control).

### Statistical analyses

The D’Agostino & Pearson test was used to determine normality. In Fig. 1C, comparison between no treatment and control EEV treatment was performed using a two-way ANOVA followed by Bonferroni’s multiple comparisons test. Comparisons of groups normalized to untreated conditions or control EEVs were performed using a Wilcoxon signed rank test with theoretical median set at 1 (Figs. 2C-D, 3C-D and 4D). When comparing more than two groups, a one-way ANOVA followed by Tukey’s multiple comparison test was performed (Figs. 2B and 4B). When data did not meet the assumption of normality, the nonparametric Kruskal-Wallis test was used to compare groups, followed by Dunn’s post-hoc test (Figs. 4C, F-G). In figures representing pairwise comparisons, data are expressed as mean ± standard error of the mean (SEM). Results are considered statistically significant when *p* < 0.05. All statistical analyses mentioned above were performed using GraphPad Prism 10.1.2 (GraphPad Software Inc., San Diego, CA, USA) software.

## Results

### BMEC-like cells internalize EEVs from healthy donors

Blood samples were collected from healthy donors, and erythrocytes were immediately isolated using standard procedures, followed by CellMask Deep Red labeling to enable the visualization of EEVs that shed from the plasma membrane. The efficiency of CellMask labeling was evaluated using FACS quantification, and 100% of cells were APC-positive (Figures S1A-B). To promote the production and release of EEVs in vitro, erythrocytes were treated with a calcium ionophore solution for one hour and EEV secretion was confirmed by quantifying the concentration of APC-positive events using a nanoparticle FACS instrument. Data show that EEVs collected from unlabeled erythrocytes are negative for APC, while EEVs secreted from pre-labeled cells are APC-positive (Figure S1E). To corroborate the vesicular nature of the events identified by the nanoparticle FACS, EEVs were incubated in a solution of 0.1% Triton X-100 to permeabilize the membranes. As a result, a reduced number of events was detected by nanoparticle FACS, validating the specificity of this approach to quantify EEVs and attest to the secretion of vesicles by erythrocytes (Figure S1E). Once the EEV production and quantification methods were established, a microscopy-based approach was used to validate that BMEC-like cells were able to internalize vesicles. First, iPSCs reprogrammed from the somatic cells of three different healthy donors were differentiated into BMEC-like cells following published protocols [3]. A time-course of EEV internalization revealed that incubation times between 24 and 48 h provided the best window for EEV observation (Figs. 1A-B), with a greater number of EEVs internalized by BMEC-like cells at the 48 h time point. Therefore, BMEC-like cells were incubated with EEVs for 48 h and processed by western blot to visualize changes to tight junction protein levels. Data revealed that EEV treatment did not alter levels of tight junction proteins such as VE-cadherin, ZO-1 or Claudin 5 (Fig. 1C).

### Caveolin-dependent endocytosis is implicated in EEV uptake by BMEC-like cells

Having confirmed that BMEC-like cells can uptake EEVs, the next step was to understand which transport mechanisms are implicated in the internalization process. To do so, BMEC-like cells differentiated from healthy control iPSCs were treated with EEVs in the presence or absence of endocytosis inhibitors. Filipin, an inhibitor of caveolin-dependent endocytosis, and recombinant Heparinase III, an inhibitor of adsorptive mediated endocytosis, were selected for this study. After a 48-h treatment, the cells were fixed and the number of internalized EEVs was quantified. We observed no change in the number of internalized EEVs after heparinase III treatment while a 55% reduction of EEV uptake was observed when BMEC-like cells were treated with filipin, suggesting that caveolin-dependent endocytosis may be implicated in EEV internalization (Figs. 2A-B). To validate this observation, a transwell-based model was used in which BMEC-like cells were plated on the insert membrane, and EEVs incubated in the upper chamber in the presence or absence of filipin for 48 h. To confirm that filipin treatment alone did not affect the ability of BMEC-like cells to form a leak-tight monolayer, the cells were treated with the molecule for 48 h followed by a barrier integrity assay. In this assay, the cell culture medium of the upper chamber was supplemented with 3 kDa dextran-TMRE, and the ratio of fluorescence intensity in the lower vs. upper compartments was calculated. The data demonstrated that the treatment did not permeabilize the BMEC-like cell monolayer (Fig. 2C), and we therefore proceeded with measuring EEV transcytosis from the upper to the lower chamber using the nanoparticle FACS. This experiment validated that inhibition of caveolin-dependent endocytosis reduced EEV transport across the BMEC-like cell monolayer (Fig. 2D).

### EEVs from PD patients alter the BBB and induce neuronal atrophy

Published literature from our group and others suggest that EEVs may contribute to PD pathology [18, 20]. In particular, we have previously reported that EEV proteomic changes enable the segregation of PD patients according to disease stage [18]. In light of the importance of EVs as signaling biovesicles and their ability to interact with endothelial cells, efforts were focused on investigating the potential contributions of EEVs derived from PD donors to BBB impairments. In particular, an experimental paradigm was established to determine whether EEVs derived from individuals with PD induce BBB deficits when the barrier is generated using control cells. To address this question, we leveraged a 3D brain chip model that we developed using a microfluidic technology, which enables the formation of fully functional endothelial vessels adjacent to human primary pericytes and iPSC-derived astrocytes [3]. Brain chips were produced using control iPSC-derived cells and the vessels perfused with EEVs derived from the erythrocytes of age and sex-matched healthy controls (*n* = 6), or donors diagnosed with mild (*n* = 5, UPDRS = 28±5) or severe PD (*n* = 5, UPDRS = 81±11) according to the UPDRS clinical scale (Figs. 3A-B). After a 48-h incubation, BBB integrity was analyzed by measuring the leakage of a 3 kDa dextran-TMRE dye from the vascular to the brain compartment of the BBB chip. EEVs generated by erythrocyte samples from PD donors with a mild or severe clinical stage increased 3 kDa dextran-TMRE leakage by 50%, indicating a weakened barrier function (Fig. 3C). Next, nanoparticle FACS was used to quantify the levels of perfused EEVs that transcytosed the vessel, and the results revealed that approximately 50% more EEVs crossed the vessel and entered the brain compartment of the model when they were derived from severe PD donors vs. controls (Fig. 3D).

To further understand the implications of these observations on endothelial cells, experiments were conducted on BMEC-like cell monolayers treated with EEVs from age- and sex-matched healthy controls or patients diagnosed with mild or severe PD for a total of 48 h. First, the data unveiled that BMEC-like cell surface area is reduced when they are grown in the presence of EEVs, regardless of control or PD status (Figs. 4A-B). Next, the number of vesicles internalized by BMEC-like cells was higher when EEVs were derived from patients with mild clinical symptoms on the UPDRS clinical scale (UPDRS = 29±4), compared to patients with severe PD symptoms (UPDRS = 80±10) or control donors (Fig. 4C). Then, changes to junction proteins were assessed, as these can impact transcytosis and mediate BBB alterations in the brain chip. Therefore, the expression levels of VE-cadherin, ZO-1 and Claudin 5 were measured after a 48-h treatment with EEVs derived from erythrocyte samples of age and sex-matched healthy controls, or from PD patients diagnosed with mild or severe clinical symptoms. Quantifications revealed that ZO-1 and Claudin 5 levels were decreased after treatment with PD EEVs, with subtle differences between iPSC lines, but VE-cadherin levels remained unchanged (Fig. 4D). The neurotoxic potential of EEVs on iPSC-differentiated dopaminergic neurons was then evaluated to determine if these vesicles could contribute to PD progression by initiating the atrophy of healthy dopaminergic neurons. Accordingly, neurons were differentiated from the iPSCs of unaffected individuals (i.e. control), exposed to a 72-h treatment with EEVs generated from the erythrocytes of PD patients or age- and sex-matched healthy controls, and neurite length measurements were used as a readout for cell health. While EEVs were not detected within neurons by immunofluorescence, ~ 25% reduction in neurite length was documented when dopaminergic (i.e. TH^+^) neurons were exposed to EEVs generated from PD donors compared to control donors. By taking advantage of our iPSC differentiation protocol that produces a mixed culture of ~ 70% dopaminergic neurons (i.e. TH^+^/MAP2^+^) and ~ 30% non-dopaminergic neurons (TH^−^ /MAP2^+^),dendrite lengths of neurons that did not express TH were measured to identify potential subtype-specific effects. Our data show contrasting results by revealing that non-dopaminergic neurons were not affected by EEVs, thus demonstrating the selective vulnerability of PD-relevant neuronal populations to EEVs (Figs. 4E-G).

## Discussion

A significant number of studies has focused on the use of plasma-derived EVs as diagnostic biomarkers of neurodegenerative disorders [18, 28–30]. These studies suggest that the contents of plasma EVs can vary over the course of disease which also implies that EV-based paracrine communication may change during pathological states. However, the ability of plasma-borne EVs to initiate or accelerate neurodegenerative diseases, such as PD, remains to be understood. In particular, the brain parenchyma is shielded from the systemic circulation by the BBB that controls the entry of molecules into the brain [31]. This regulation involves numerous endothelial transporters that mediate the transcytosis of blood-borne components, and underlines the specificity of molecular exchanges at the BBB [31, 32]. Additionally, these different aspects of BBB integrity can be independently regulated, implying the importance of determining how blood-borne EVs cross the endothelium, and if this aspect of the BBB becomes less restrictive as neurodegenerative disorders progress from mild to more severe states. In the present study, the effects of EVs secreted by erythrocytes - previously suggested to adopt a different protein signature with disease stage in PD [18] - on the neurovascular unit were explored using a new human iPSC-based BBB modeling platform. The iPSC-based models confirmed that human EEV transcytosis occurs via caveolin-dependent endocytosis, and PD EEVs alter the molecular composition of BMEC-like cells, impact BBB integrity, and selectively affect dopaminergic neurons.

The BBB has long been considered a static barrier between the blood and the brain, but studies increasingly show that it is in fact a dynamic interface that responds to exogenous cues and changes over the course of aging [33, 34]. Published reports have explored the transport mechanisms by which EVs cross the BBB in various disease models, and data consistently support the implication of active endocytic transfer initiated at the endothelial membrane [20, 35–37]. However, while receptor-mediated endocytosis appears to be a common mechanism, the dominant endocytic pathway seems to vary depending on the nature of the EVs under investigation [36]. In our study of EEVs derived from healthy donors, caveolin-dependent endocytosis emerged as the primary transport mechanism, but other types of EVs can navigate the BBB through caveolin-independent mechanisms, as shown for tumor-derived EVs [35]. Our data was collected in a humanized BBB model with high translatability to patients, and the validity of these in vitro findings are strengthened by a complementary study that also reported EEV transport across the BBB [20]. In this latter study, EEV entry into the brain was exacerbated by intraperitoneal lipopolysaccharide (LPS) injections [20], which reduced trans-endothelial electrical resistance (TEER) and induced pro-inflammatory conditions.

EV composition mirrors the health or disease status of the parent cell, and we previously showed that the molecular profile of EEVs reflects stratification of PD patients by clinical score [18]. Given that EVs can serve as cargos of biological signals, we exploredthe possibility that EEVs generated by the erythrocytes of PD patients may display detrimental functions at the BBB and gain a greater ability to penetrate the brain. To address this question, a 3D BBB chip model was produced using control iPSC-derived cells, and changes to BBB function and transcytosis were measured. The advantage of this model, besides the formation of a 3D blood vessel, is the presence of fluid sheer stress that recapitulates a mechanical stimulus central to BMEC function, that differentiates a luminal vs. abluminal compartment [38, 39]. When perfused with EEVs produced from mild or severe PD patients vs. age and sex-matched healthy controls, vessel-forming BMEC-like cells altered their physical and molecular barrier function. Notably, barrier alteration and increased EEV levels in the brain compartment of PD-treated BBB chips was observed, along with reduced ZO-1 and Claudin 5 protein levels in BMEC-like cells. In addition, when dopaminergic neurons differentiated from control iPSCs were treated with EEVs, neuronal atrophy was measured after mild and severe PD EEVs treatment. This finding is highly relevant to PD etiology because dopaminergic neurons are the most vulnerable population affected by the disease. Using confocal microscopy to visualize EEV uptake by neuronal monocultures, and as conducted for the BMEC-like cells, neurons did not appear to have internalized vesicles. While additional experiments are needed to understand how EEVs triggered neuron atrophy, it is possible that neurons did not internalize EEVs via endocytosis but engulfed them via a process of fusion with the plasma membrane, or that EEVs acted extracellularly via neuronal membrane receptors [40, 41]. Another important finding is that control EEVs induced neurite shortening, albeit to a lower extent than PD EEVs. It suggests that BBB weakening and/or increased EEV transcytosis, regardless of disease status, could be detrimental to dopaminergic neurons. Of relevance, our previous studies did not detect the presence of the PD-related α-Syn protein in EEVs generated from our library of erythrocytes [18], but others have suggested that α-Syn can be found within EEVs. Hence, the relationships between erythrocytic α-Syn and PD pathology remains to be fully explored [20, 42, 43].

A strength of our study is that these results were generated using human iPSC-derived cells, which allow the investigation of EV permeability across the BBB in an experimental human-related system. Here, we restricted our investigations to a control BBB in order to characterize the interactions between EEVs and the BBB in healthy conditions. In doing so, any loss of BBB function or reported toxicity can be directly related to the disease status of the EEVs, eliminating potential confounding factors that could arise from a diseased BBB. In fact, the use of a control BBB model could be considered a pre-PD or prodromal PD stage and enable the mechanistic investigation of early events that contribute to full-blown phenotypes. Notably, the observation that PD EEVs weaken BBB integrity and reduced endothelial tight junction levels might suggest that potentially toxic blood-borne molecules could infiltrate the brain parenchyma and further exacerbate and accelerate neuroinflammation and neurodegeneration. While this is an emerging field, published studies support the hypothesis of increased extravasated erythrocytes and serum proteins into the brain of individuals with PD [6, 44]. Therefore, future research could build on these findings and evaluate how a PD-derived BBB model may affect EEV endocytosis and toxicity.

Glial cells, particularly microglia and astrocytes, play a crucial role in the brain’s response to injury and inflammation, and their inclusion in BBB models is important when studying the consequences of EEV entry into the brain parenchyma. Here, the 3D BBB model included iPSC-derived astrocytes, as these cells are essential to the formation and maintenance of the BBB. Given that astrocytes and microglia play pivotal roles in PD by supporting neuronal health, regulating neuroinflammation, and sustaining the integrity of the BBB, their dysfunction contributes to the progressive degeneration of dopaminergic neurons, worsening PD pathology [3, 17, 45, 46]. While microglia were not included in this model, they would be an important addition to future experiments, as reports suggest that transcytosed EEVs may activate microglia [20]. Future studies could also explore the impact of EEVs on other glial cells (e.g. astrocytes and mural cells) to further understand their role in the disease process. Of note, animal studies suggest that intravenous injection of EEVs from PD patients promote α-Syn oligomerization in the striatum, where astrocytes and microglia uptake higher proportions of EEVs compared to neurons [47]. The presence of α-Syn within EEVs has proven more contentious in published studies, and it is still unclear whether the phenotypes observed in [47] are directly mediated by α-Syn enclosed within EEVs. A report by Yang and colleagues supports a pathological role for α-Syn-EEVs at other physiological barriers, as it was shown that these vesicles can also weaken the gut vasculature and transport α-Syn into the gastrointestinal tract [48]. Overall, additional work is needed to pinpoint the contributions of each EEV molecular component to potential PD-related features, explore the diversity of biochemical signals that may drive disease phenotypes, and define the implication of the aging process in these phenotypes. Such studies could take advantage of EEV proteomics data, such as those published by Lamontagne-Proulx and colleagues [18], that list differentially regulated proteins in control vs. PD EEVs. For example, Nicotinamide adenine dinucleotide (NAD) was found at lower levels in EEVs derived from erythrocyte samples of PD donors vs. controls. This protein is a coenzyme implicated in metabolic reactions and biological processes relevant to PD, among which mitochondrial respiration, cellular senescence or immune homeostasis [49, 50]. The relevance of this protein as a protective factor is further demonstrated by publications considering NAD replenishment therapies as potential clinical strategies for people with PD [51, 52]. In addition, age can significantly affect the integrity and function of the BBB. As individuals age, transport across the BBB is altered, which may impact the neuronal microenvironment and allow systemic substances to enter the brain and contribute to neuroinflammation [53]. This increased permeability can result from changes to endothelial cell function, tight junction protein expression, and overall vascular health, and has been documented in neurodegenerative disorders, including PD [2, 54]. While this study documented that EEVs from young healthy donors transcytose into the brain chip via a caveolin-dependant mechanism, additional investigations could explore how the age of the donors may impact the transcytosis pathways implicated in EEV transport across the BBB, in both physiological and disease conditions. Furthermore, complementary studies in animal models could assess the impact of PD-derived EEVs in young vs. old wild-type and PD mouse models and, in doing so, further document the implication of peripheral molecules to PD pathology.

In conclusion, our data support the potential contributions of erythrocytes, and in particular EEVs, to neuropathology documented in the brain of PD patients. Our approach leveraged advanced BBB modeling technologies using organ-on-chip platforms, which mimic the complex cellular architecture of the neurovascular unit and enables the precise quantification of blood-brain exchanges in a humanized model. Combined, this work supports a model of periphery-driven BBB alteration and potential peripheral contribution to dopaminergic neurodegeneration in PD.

## Electronic supplementary material

Below is the link to the electronic supplementary material.

Supplementary Material 1

## Data Availability

Data is provided within the manuscript or supplementary information files.
