# Supplementary material for: Erythrocyte-derived extracellular vesicles transcytose across the blood-brain barrier to induce Parkinson’s disease-like neurodegeneration
Source: Fluids Barriers CNS. 2025 Apr 14;22:38. doi: 10.1186/s12987-025-00646-9 (PMC11998243; doi:10.1186/s12987-025-00646-9)
Supplement: Supplementary file 1 — Supplementary Material 1 [file 12987_2025_646_MOESM1_ESM.docx]

SUPPLEMENTARY MATERIAL

Erythrocyte-derived extracellular vesicles transcytose across the blood-brain barrier to induce Parkinson's disease-like neurodegeneration

Hélèna L. Denis^1,2*^, Aurélie de Rus Jacquet^1,2*^, Melanie Alpaugh^1,2,3^, Michel Panisset^4^, Roger A Barker^5^, Éric Boilard^1,6^, Francesca Cicchetti^1,2^.

** equal contribution*

^1^ Centre de recherche du CHU de Québec, Québec, QC, Canada; ^2^ Département de Psychiatrie & Neurosciences, Université Laval, Québec, QC, Canada; ^3^ Department of Molecular and Cellular Biology, University of Guelph, Guelph (current affiliation) ; ^4^ Centre Hospitalier de l'Université de Montréal and Centre de recherche du Centre Hospitalier de l'Université de Montréal, Hôpital Notre-Dame, Département de médicine, Université de Montréal, Montréal, QC, Canada; ^5^ Department of Clinical Neurosciences, John van Geest Centre for Brain Repair, University of Cambridge, Cambridge, United Kingdom; ^6^ Département de microbiologie et immunologie, Université Laval, Québec, QC, Canada

**Correspondence:**

Francesca Cicchetti, Ph.D.

Centre de Recherche du CHU de Québec

Axe Neurosciences, T2-07

2705, Boulevard Laurier

Québec, QC, G1V 4G2, Canada

Tel #: (418) 656-4141 ext. 48853

Fax #: (418) 654-2753

E-mail: francesca.cicchetti@crchudequebec.ulaval.ca


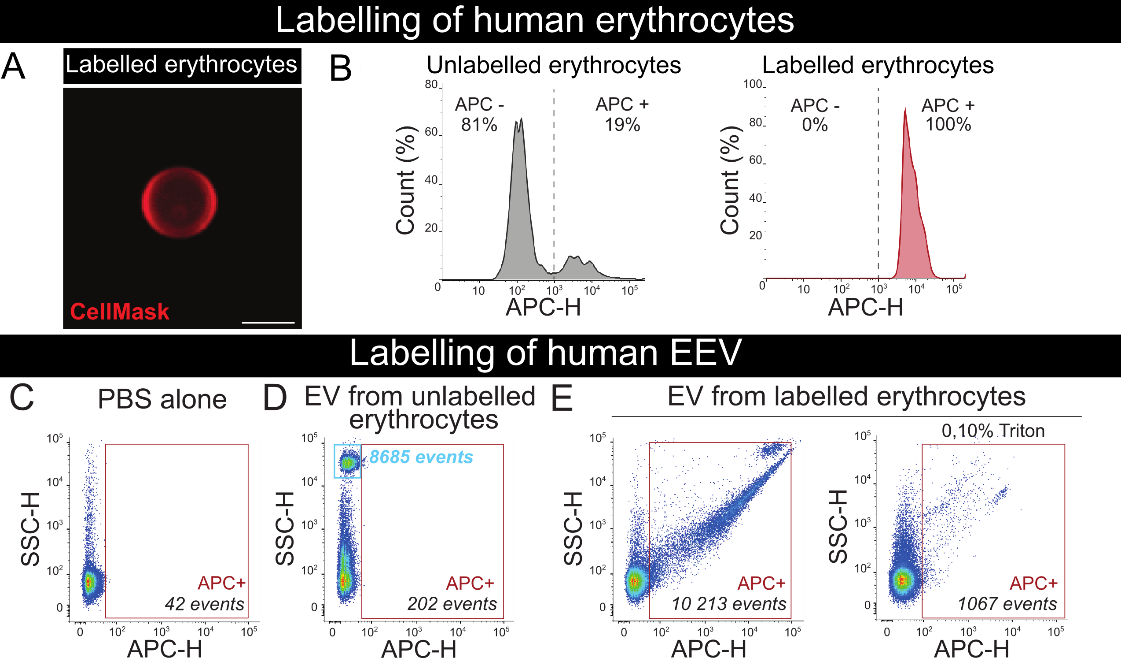


**Supplementary Figure 1. Validation of EEV labeling and detection.** (**A**) Confocal image of an erythrocytes stained with CellMask Deep Red. Scale bar: 5 μm. (**B**) Representative FACS-based quantification of the proportion of APC-positive erythrocytes after staining with CellMask Deep Red. (**C-E**) Representative nanoparticle FACS-based quantification of EEVs produced after activation of erythrocytes with calcium ionophore. No positive events were detected in the PBS negative control (**C**), unlabelled erythrocytes produced unstained APC-negative EEVs (blue box) (**D**), and EEV derived from CellMask Deep Red-labeled erythrocytes displayed abundant APC+ events which were eliminated after incubation with 0.1% Triton (**E**). *Abbreviations:* APC-H, Allophycocyanin height; EEV, extracellular vesicles derived from erythrocytes; SSC-H, side scatter height.
